# Supplementary material for: Bee Venom Proteins Enhance Proton Absorption by Membranes Composed of Phospholipids of the Myelin Sheath and Endoplasmic Reticulum: Pharmacological Relevance
Source: Pharmaceuticals (Basel). 2025 Sep 5;18(9):1334. doi: 10.3390/ph18091334 (PMC12472953; doi:10.3390/ph18091334)
Supplement: Supplementary file 1 [file pharmaceuticals-18-01334-s001.zip › pharmaceuticals-3825278-supplementary.pdf]

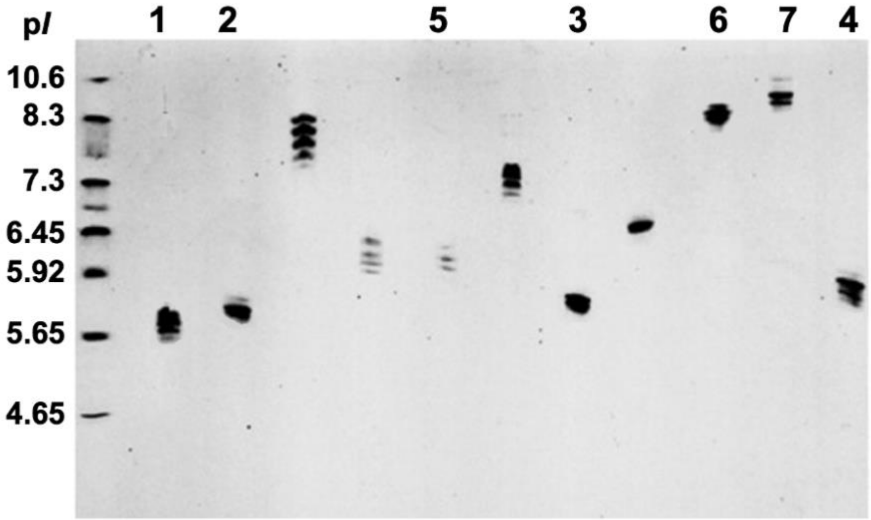

Supplementary Figure S1. Isoelectric focusing (IEF) of seven bee venom fractions and four unrelated samples on Ready Gel Precast Gels with ampholytes spanning a pH gradient of 3.0–10.5. Lanes representing bee venom fractions are numbered at the top of the gel, while lanes for unrelated samples remain unmarked. The unmarked lane on the far left contains standard p*I* markers (FMC Corporation, Rockland, ME, USA) with a p*I* range of 4.65–10.6.
